# Biophysical and functional characterization of the N-terminal domain of the cat T1R1 umami taste receptor expressed in *Escherichia coli*

**DOI:** 10.1371/journal.pone.0187051

**Published:** 2017-10-30

**Authors:** Christine Belloir, Jimmy Savistchenko, Fabrice Neiers, Andrew J. Taylor, Scott McGrane, Loïc Briand

**Affiliations:** 1 Centre des Sciences du Goût et de l'Alimentation, INRA, CNRS, Bourgogne Franche-Comté University, AgroSup Dijon, Dijon, France; 2 WALTHAM Centre for Pet Nutrition, Melton Mowbray, Leicestershire, Great Britain; The University of Tokyo, JAPAN

## Abstract

Umami taste perception is mediated by the heterodimeric G-protein coupled receptors (GPCRs), formed by the assembly of T1R1 and T1R3 subunits. T1R1 and T1R3 subunits are class C GPCRs whose members share common structural homologies including a long N-terminal domain (NTD) linked to a seven transmembrane domain by a short cysteine-rich region. The NTD of the T1R1 subunit contains the primary binding site for umami stimuli, such as L-glutamate (L-Glu) for humans. Inosine-5’-monophosphate (IMP) binds at a location close to the opening of the T1R1-NTD “flytrap”, thus creating the observed synergistic response between L-Glu and IMP. T1R1/T1R3 binding studies have revealed species-dependent differences. While human T1R1/T1R3 is activated specifically by L-Glu, the T1R1/T1R3 in other species is a broadly tuned receptor, sensitive to a range of L-amino acids. Because domestic cats are obligate carnivores, they display strong preferences for some specific amino acids. To better understand the structural basis of umami stimuli recognition by non-human taste receptors, we measured the binding of selected amino acids to cat T1R1/T1R3 (cT1R1/cT1R3) umami taste receptor. For this purpose, we expressed cT1R1-NTD in bacteria as inclusion bodies. After purification, refolding of the protein was achieved. Circular dichroism spectroscopic studies revealed that cT1R1-NTD was well renatured with evidence of secondary structures. Using size-exclusion chromatography coupled to light scattering, we found that the cT1R1-NTD behaves as a monomer. Ligand binding quantified by intrinsic tryptophan fluorescence showed that cT1R1-NTD is capable of binding L-amino acids with *K*_d_ values in the micromolar range. We demonstrated that IMP potentiates L-amino acid binding onto renatured cT1R1-NTD. Interestingly, our results revealed that IMP binds the extracellular domain in the absence of L-amino acids. Thus, this study demonstrates that the feasibility to produce milligram quantities of cT1R1-NTD for functional and structural studies.

## Introduction

Umami is one of the five basic taste qualities whose proposed physiological role is to signal the presence of proteins in food by detecting free L-amino acids. In humans, this typical taste is elicited mainly by L-glutamic acid (L-Glu), which is found naturally in many protein-rich foods such as meat, poultry, fish and seafood. Comparative behavioural studies have demonstrated widely divergent taste preference to L-amino acids across animal species, such as mice [1], rats [2], rhesus monkey [3] and domestic pigs [4–6]. For instance, only two amino acids, L-Glu and, to a lesser extent, L-aspartic acid (L-Asp) are umami stimuli for humans [7]. In contrast, behavioural and electrophysiological experiments have revealed that rodents are able to perceive a larger range of L-amino acids [8]. A unique feature of umami taste is its potentiation by purine nucleotides such as inosine-5’-monophosphate (IMP) and guanosine-5’-monophosphate (GMP), which also elicit some umami taste on their own [9].

The umami taste receptor is a heterodimeric receptor composed of the T1R1 and T1R3 subunits, whereas T1R2 and T1R3 subunits assemble to form the sweet taste receptor [7, 10]. T1R1, T1R2 and T1R3 subunits belong to the family of class C, G protein-coupled receptors (GPCRs). All the members of this family share a similar architecture constituted by a large N-terminal domain (NTD) linked to the characteristic heptahelical transmembrane domain (HD) by a cysteine-rich region (CRR). It has been shown that the NTD of T1R1 (T1R1-NTD), also described in the literature as the Venus flytrap domain, which has sequence homology to bacterial periplasmic amino acid binding proteins, contains the orthosteric binding site for umami molecules [11, 12]. Structural analysis of the homodimeric rat mGluR1-NTD revealed that the extracellular ligand-binding domain is made by two lobes separated by a cleft in which L-Glu binds. The crystal structure solved in the absence and presence of L-Glu demonstrated that mGluR1-NTD exists in dynamic equilibrium between an open and closed conformation, which is stabilized by the bound L-Glu [13]. Recently, Nuemket et al. [14] deciphered the X-ray structure of medaka fish T1R2-NTD/T1R3-NTD heterodimer bound to different amino acids, preferable taste substances to these fish. The authors characterized the binding sites of the receptor and found a compact heterodimer arrangement in the presence of amino acid ligands.

Cellular assays combined with site-directed mutagenesis have revealed the molecular mechanism of synergy between L-Glu and 5’ribonucleotides. Thus, it has been demonstrated that L-Glu binds in the hinge region of the T1R1-NTD inducing its closure whereas IMP binds to an adjacent site, close to the opening, further stabilising the closed conformation of the T1R1-NTD [12]. More recently, the key residues responsible for the species difference between human and mouse T1R1/T1R3 binding selectivity have been identified [15]. Using human-mouse chimera, site-directed mutagenesis and molecular modelling, the authors demonstrated a complex mechanism of L-amino acid recognition involving both the orthosteric and non-orthosteric site of T1R1. There are multiple lines of evidence arguing for the presence of additional umami receptors in mice [16–19], including truncated forms of metabotropic glutamate receptors types 1 (mGluR1) and 4 (mGluR4) [20, 21]. However, taste-mGluRs are activated by some L-amino acids and analogs, but are not reported to be sensitive to ribonucleotides (i.e. acting as agonists or positive modulators).

The domestic cat (*Felis catus*) is an obligate carnivore and naturally eats a high protein, low carbohydrate diet. Cats respond positively to salty molecules and are known to avoid stimuli that taste sour or bitter for humans. Recently, the functional analysis of feline bitter taste receptors using cellular based assays has been reported [22, 23]. Physiological and behavioural studies have demonstrated that domestic cats are sensitive to various L-amino acids [24–27]. Molecular modelling of cat T1R1 and *in vivo* tests have revealed that, contrary to the human umami receptor where the acidic amino acids (L-Glu and L-Asp) are the key ligands, the cat receptor has a broadly tuned binding specificity towards various amino acids of different classes, including L-histidine (L-His) and L-alanine (L-Ala) [26]. In addition, this study revealed that synergism between 5’-monophosphate ribonucleotides, such as IMP and L-amino acids exists in cats [26, 28], as previously demonstrated in human and mouse umami receptors [9, 29]. Surprisingly, it has been shown that cats display indifference toward sugars or artificial sweeteners, whereas the other taste modalities in the cat are functional. The open reading frame of the gene coding the T1R2 subunit is a pseudogene (non functional and non-expressed gene) and accounts for insensitivity of cats toward sugars [30]. In contrast, Giant pandas lost the T1R1 gene, which is non-functional, in accordance with their herbivore diet comprised almost exclusively of bamboo [31, 32]. Intriguingly, a recent study revealed an alternative T1R2-independent mechanism for carbohydrate detection in hummingbirds. The authors proposed that the ancestral hummingbird umami T1R1/T1R3 receptor evolved to function as a sugar receptor allowing these birds to perceive sweet nectar [33]. In cats, the T1R1 gene is functional [30] and it was reported that T1R1 assembles with T1R3 forming the heterodimer T1R1/T1R3, which is sensitive to some L-amino acids [26]. In addition, human and rodent T1R3, unlike T1R1, seems to have the ability to form T1R3/T1R3 homodimer sensitive to sugars, but only at high concentrations [10, 34]. In cats, the function of the homodimer of cT1R3 receptor remains unknown.

Ligand binding studies of the isolated mouse and human T1R2- and T1R3-NTDs expressed in *Escherichia coli* have shown that T1R-NTDs are able to bind natural sugars (sucrose and glucose) and the chlorodeoxysugar sucralose with distinct and physiological relevant affinities [35–37]. To further understand the structural basis of L-amino acid recognition by the umami taste receptor and the molecular mechanism of IMP synergy in ligand binding, we report here the expression of the N-terminal domain of the cat T1R1 taste receptor (cT1R1-NTD) in *Escherichia coli* as aggregated inactive proteins called inclusion bodies. The expression of target proteins using this approach has several advantages including the very high yield of produced protein, the easy isolation of the inclusion bodies with a high degree of purity and the protection of the expressed protein from proteolysis. Pure cT1R1-NTD inclusion bodies were solubilised and refolded *in vitro*. Circular dichroism (CD) spectroscopic studies revealed that cT1R1-NTD was properly refolded with evidence of secondary structures. Using size-exclusion chromatography coupled to multiangle laser light scattering (SEC-MALS), we found that the cT1R1-NTD behaved as a monomer. Using intrinsic tryptophan fluorescence, we demonstrated that cT1R1-NTD was able to bind six selected L-amino acids with distinct and physiological relevant affinity. In addition, we demonstrated the positive effect of IMP on L-amino acid binding, further demonstrating that refolded cT1R1-NTD is functional. Importantly, we observed that IMP is able to bind to the extracellular domain in the absence of L-amino acid. This study offers interesting new experimental strategies to screen new tastants or to provide new insights into molecular determinants of umami perception including NMR spectroscopic analyses or crystallization trials.

## Materials and methods

### Construction of the expression plasmid

**Fig 1:**
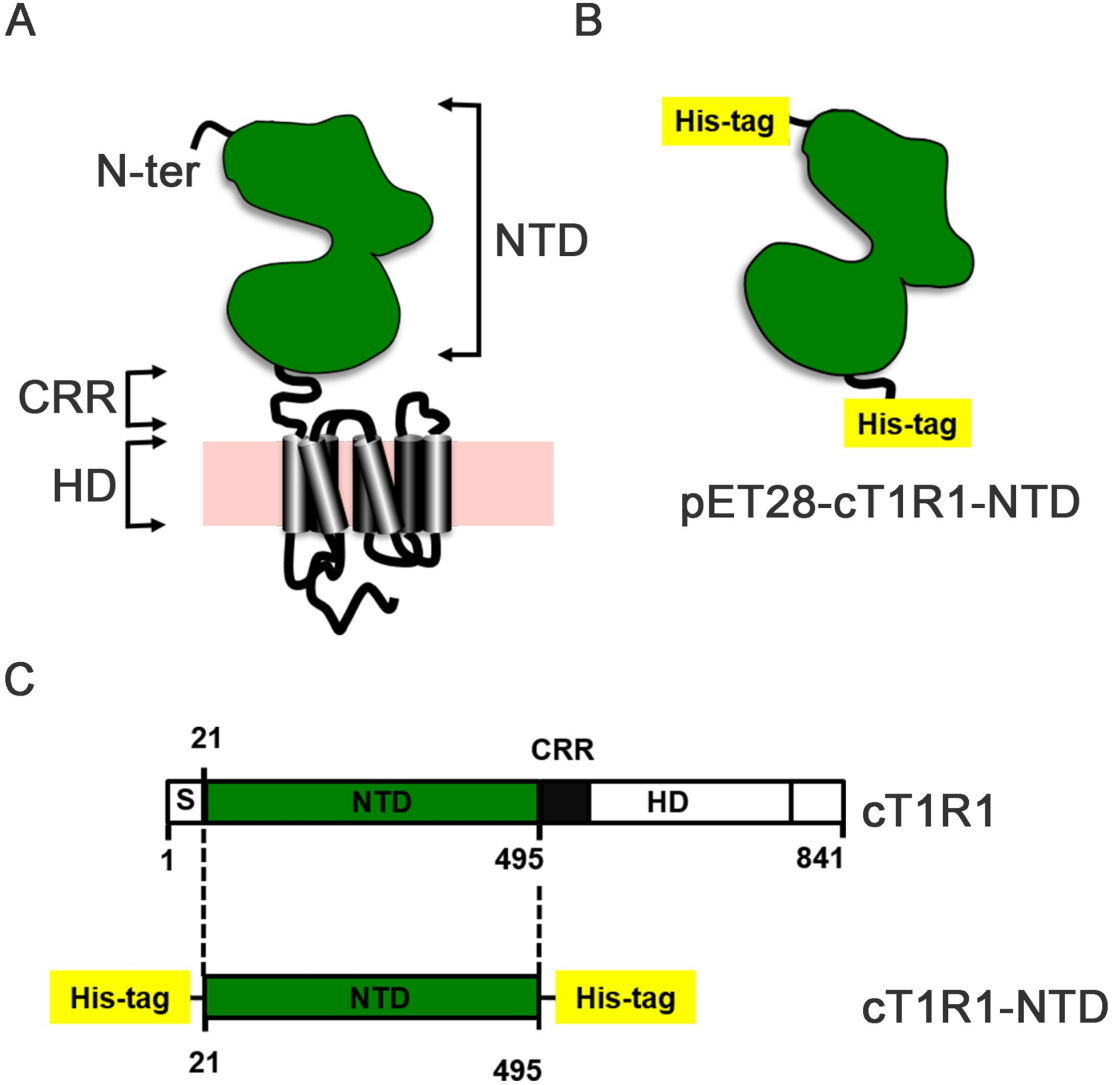
Strategy used for expression of the cat T1R1-NTD in bacteria. (A) The N-terminal domain (NTD) of cT1R1 was expressed independently from the transmembrane heptahelical domain (HD), minus a short putative signal peptide (S), and a cysteine-rich region (CRR). (B) The pET28-cT1R1-NTD plasmid encodes a fusion protein that contains an N-terminal His-tag that can be cleaved with thrombin, followed by cT1R1-NTD (Leu21-Ser495) and a C-terminal His-tag. (C) Full-length cT1R1 is presented according to its primary amino acid sequence deduced from DNA sequence. The numerical positions of amino acid residues of cT1R1 are indicated.

The cDNA sequence encoding cT1R1-NTD minus a putative signal sequence and the cysteine-rich region (CRR) was optimized for expression in *E*. *coli* and synthesized by GeneArt^®^ Gene Synthesis (Life Technologies). The synthetic cDNA of cT1R1-NTD was subcloned into the *Nde*I and *Eco*RI restriction sites of pET28a (Novagen) expression vector as described for human T1R1-NTD [38]. The resulting expression vector pET28-cT1R1-NTD encodes a fusion protein comprising a N-terminal (His)_6_-tag, followed by the NTD and a C-terminus (His)_6_-tag (Fig 1 and S1 Fig).

### Recombinant protein expression and inclusion bodies preparation

For production of cT1R1-NTD, *E*. *coli* BL21 (DE3) cells (Stratagene) containing pET28-cT1R1-NTD plasmid were grown in 2xYT medium (16 g/l tryptone, 10 g/l yeast extract, 5 g/l NaCl, pH 7.5) supplemented with kanamycin (45 μg/ml) at 37°C and induced to an optical density of 0.8 at 600 nm. Protein overexpression was induced for 3 h at 20°C by adding 0.1 mM isopropyl-β-D-thiogalactoside (IPTG). Cells were harvested by centrifugation (4500 x *g* for 15 min at 4°C) and stored at -20°C until use. Inclusion bodies of cT1R1-NTD were purified as previously described [35]. Briefly, cells from 2 litres of culture were re-suspended in 50 ml of ice-cold lysis buffer (50 mM Tris-HCl pH 8.0, 10 mM EDTA, 100 mM NaCl, 0.5% (v/v) Triton X-100, 1 mM PMSF, DNase I, 3 mM desoxycholic acid), and disrupted by sonication. After centrifugation (10,000 x *g* for 15 min, 4°C), the pellet containing inclusion bodies was thoroughly re-suspended in 100 ml of 50 mM Tris-HCl, pH 8.0, 10 mM EDTA, 500 mM NaCl, 0.5% (v/v) Triton X-100 and disrupted by sonication, followed by a second centrifugation. Inclusion bodies were washed twice using 200 ml of 50 mM Tris-HCl pH 8.0, 10 mM EDTA, 150 mM NaCl, 0.5% (v/v) Triton X-100, 4 M urea. The inclusion bodies were then washed with 100 ml of water and dissolved in 40 ml of solubilizing buffer (50 mM Na_2_HPO_4_ pH 8.0, 0.5 M NaCl, 6 M guanidine hydrochloride, 25 mM dithiothreitol (DTT)) and stirred for 12 h at room temperature. Protein purity and integrity were verified by SDS-PAGE. Protein solutions were clarified by filtration using 0.45 μm filters followed by centrifugation (10,000 x *g* for 45 min, 10°C), concentrated to 20 mg/ml using Amicon concentrator (12–14 kDa molecular mass cut-off) and stored at -20°C. Protein concentration was determined by measuring absorbance at 280 nm with a molar extinction coefficient of 77,975 L.mol^-1^.cm^-1^.

### In vitro refolding of cT1R1-NTD

cT1R1-NTD was refolded by dialysis using the protocol developed for hT1R3-NTD [35] with slight modifications (S2 Fig). Twenty milligrams of pure inclusion bodies were diluted into 25 mM Tris-HCl pH 8.0, 8 M urea, 12.5 mM DTT, 20 mM sodium dodecyl sulfate (SDS), 0.1mM n-dodecyl-β-D-maltoside (DDM) to obtain a final protein concentration of 50 μg/ml and incubated at 50°C in a water-bath for 1 h. Protein solution was then successively dialysed (12–14 kDa molecular mass cut-off) for 16 h against 3.2 l of 50 mM Tris-HCl pH 8.0, 1 M urea, 1 mM EDTA, 1mM DTT, 20 mM SDS, 0.1 mM DDM then for 2 h against 3.2 l of 50 mM Tris-HCl pH 8.0, 1 mM DTT, 2 mM SDS, 0.1 mM DDM at room temperature. After filtration through 0.45 μm pore size filter and concentration to a final protein concentration of 0.5–1 mg/ml using a Vivaspin concentrator (50 kDa molecular mass cut-off), cT1R1-NTD refolding was initiated by precipitating dodecyl sulfate as its potassium salt, adding KCl in the presence of DDM (5 mM final concentration) and 300 mM NaCl. Under vigorous stirring, KCl (2 M) was added to the protein solution in order to bring the nominal concentration of free K^+^ ions to 300 mM. After 30 min, the SDS precipitate was removed by centrifugation at 48,400 x *g* for 1 h at 15°C. To remove residual dodecyl sulfate, the supernatant was extensively dialysed (50 kDa molecular mass cut-off) against 50 mM Tris-HCl pH 8.0, 150 mM KCl, 150 mM NaCl, 1 mM DTT, 0.1 mM DDM (~total dialysis time: 16 h) at room temperature. Refolded cT1R1-NTD was concentrated 10 fold (2-5mg/ml) using a Vivaspin concentrator (50 kDa molecular mass cut-off). After filtration through 0.22 μm pore size filter to remove possible aggregates, cT1R1-NTD was purified using size exclusion chromatography (SEC). SEC was performed through a gel filtration chromatography column (Superdex200 high performance, 10 mm i.d. x 300 mm; GE Healthcare) connected to a FPLC ÄKTA pure system (GE Healthcare). The column was equilibrated at 0.5 ml/min with 50 mM Tris-HCl pH 8.0, 150 mM NaCl, 1 mM DTT, 0.1 mM DDM. Two or three injections of 0.5 ml of cT1R1-NTD were performed. The purity of the sample was assessed by SDS-PAGE [39], using Coomassie staining.

To detect cT1R1-NTD, protein samples were separated by SDS-PAGE, transferred, and immunoblotted with mouse anti-his-tag primary antibody (diluted 1:2,000, Bio-Rad) followed by goat anti-mouse horseradish peroxidase-conjugated secondary antibody (diluted 1:50,000, Bio-Rad). Immunoblots were detected using ECL chemiluminescent kit (Clarity Western ECL Substrat, Bio-Rad) and ChemiDoc apparatus (Bio-Rad). Eluted protein peak fractions that corresponded to monomeric protein were collected and pooled. The protein concentration was determined by measuring absorbance at 280 nm.

### Protein identification

Purified protein band sequences were identified by peptide mass fingerprinting involving tryptic cleavage, combined with MALDI-ToF analysis. Protein identification was carried out at the PAPPSO platform (INRA, Jouy-en-Josas, France) using MS-Fit (Protein Prospector, UCSF) program.

### Size Exclusion Chromatography - Multi-Angle laser Light Scattering (SEC-MALS) analysis of the refolded protein

Protein oligomeric state was studied using a Jasco PU-2080 Plus system consisting of a pump, a vacuum degasser, an autosampler, and a Silica Gel KW804 column (Shodex). Detection was performed using a triple-angle light scattering detector (Mini- DAWN^™^ TREOS, Wyatt Technology), a Shimadzu refractometer (RID-10A) and a SpectraSeries UV100 detector. Molecular weight determination was performed using the ASTRA VI software (Wyatt Technology) using a dn/dc value of 0.185 ml/g. The column used for the SEC-MALS analysis was equilibrated with SEC buffer (50 mM Tris-HCl pH 7.5, 300 mM NaCl, 0.1 mM DDM, filtered through 0.22 μm pore size filters (Millipore) at 0.5 ml/min. One hundred microliters of protein solution (10 μM) was injected.

### Circular dichroism

Circular dichroism (CD) spectra were recorded using a JASCO J-815 spectropolarimeter equipped with a Peltier temperature control. Using a 0.01-cm path length quartz cell (Hellma), protein samples (~ 0.2 mg/ml in 50 mM Tris-HCl pH 8.0, 150 mM NaCl, 1 mM DTT, 0.1 mM DDM) were recorded between 190 and 260 nm at 25°C. The spectra were corrected for buffer contributions and converted to mean residue ellipticity in deg cm^2^ dmol^-1^. Spectra were averaged over 5 scans accumulated at 0.5 nm intervals with 50-nm/min scan speed and 5-s response times. They were smoothed using the Stavitzky-Golay convolution filter with a span of 5. The secondary structure proportions were estimated using the deconvolution K2D algorithm on DichroWeb program (http://dichroweb.cryst.bbk.ac.uk/html/home.shtml) [40].

### Ligand binding assay and data analysis

The intrinsic fluorescence of cT1R1-NTD was measured as previously described by Nie et al. [37] using a Cary Eclipse spectrofluorometer (Varian Instruments) equipped with a Peltier temperature control unit. Sample proteins were excited at 295 nm and emission spectra were recorded from 300 to 400 nm, with 5-nm and 10-nm bandwidth for emission and excitation, respectively. For titration experiments, fluorescence spectra were recorded using 0.5 μM protein solution in 50 mM Tris-HCl pH 8.0, 150 mM NaCl, 1mM DTT and 0.1 mM DDM. Ligand solutions were freshly prepared in the same buffer. Successive aliquots of potential ligands were added to 400 μL of cT1R1-NTD solution. The temperature was kept constant at 20°C. Fluorescence measurements were corrected for dilution, bleaching and nonspecific buffer quenching. *K*_d_ values and standard errors were calculated from a plot of the ratio between the fluorescence intensity variation and the maximum of fluorescence intensity variation versus concentration of total ligand, obtained with a standard non-linear regression method and using one site saturation ligand binding equation using SigmaPlot software. To measure the synergism between L-amino acids and IMP, the intensities of fluorescence of cT1R1-NTD were normalized to 0% in presence 0.3 μM IMP alone and 100% at highest concentrations of L-amino acids in absence of IMP. The *K*_d_ values of ligand-receptor interactions were determined using Eq (1)

$$\frac{\Delta F}{\Delta Fmax}\;=\;\frac{B_{max}\times [L]}{{(K}_{d}+[L])}$$ (1)

Where *K*_*d*_ value is the apparent dissociation constant, ΔF is the difference between the fluorescence intensity at a given concentration of ligand and the fluorescence intensity in the absence of ligand, and ΔFmax is the difference at infinite ligand concentration [L], *B*_*max*_ is the maximum fluorescence signal, [L] corresponds to the concentration of ligands. The reported *K*_d_ values are the average of more than nine measurements performed on at least three independently refolded protein samples. IMP and L-amino acids were obtained from Sigma-Aldrich. Statistical analyses: Dunnett’s test was used to determine the statistical significance between data means.

## Results

### Bacterial expression of cT1R1-NTD

**Fig 2:**
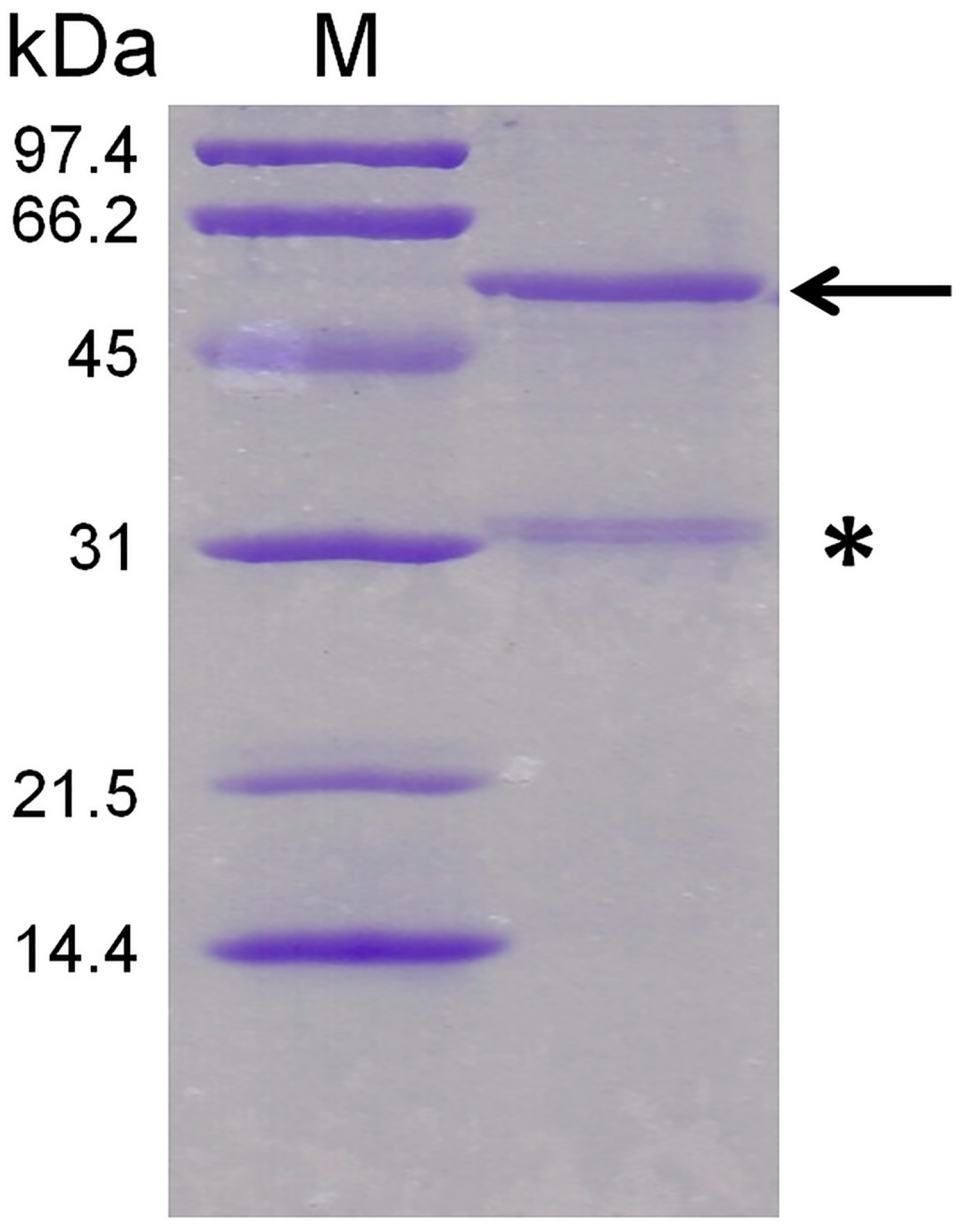
SDS-PAGE analysis of purified cT1R1-NTD inclusion bodies. cT1R1-NTD is indicated with an arrow while the star indicates a band corresponding to a N-terminal fragment of cT1R1-NTD. The proteins were separated by 12% SDS-PAGE and stained with Coomassie blue. The molecular mass markers are in lane M.

**Fig 3:**
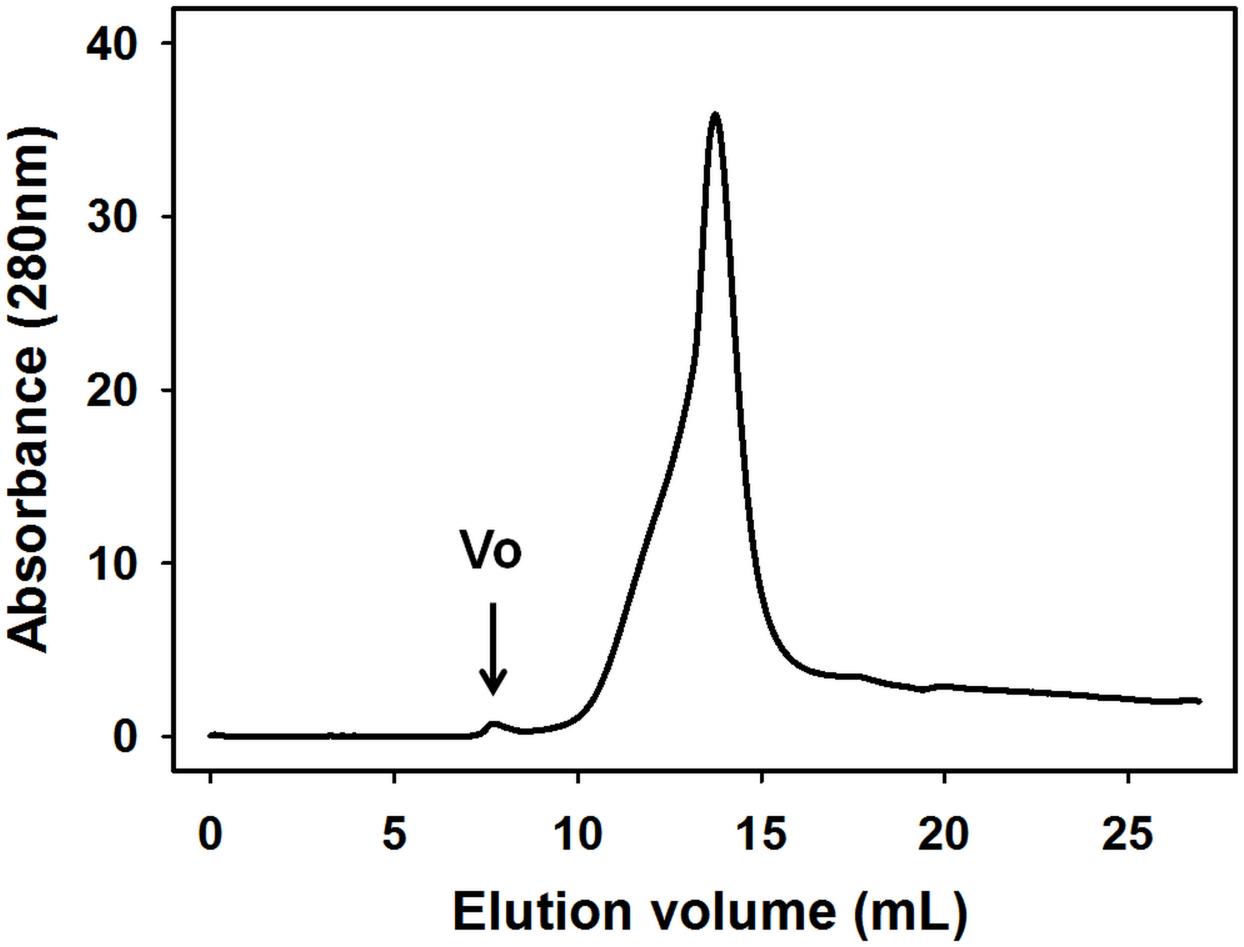
Preparative gel filtration chromatography of refolded cT1R1-NTD. Gel filtration was performed using a Superdex 200 column (10 x 300 mm, 24 mL). The column was equilibrated in 50 mM Tris-HCl pH 8.0, 150 mM NaCl, 1mM DTT, 0.1 mM DDM. The arrow indicates the position of the void volume (Vo).

Recently, we reported a simple and robust protocol to produce pure and homogenous human T1R3-NTD expressed at milligram levels using *Escherichia coli* [35, 36] following the strategy presented in Fig 1. Biophysical studies demonstrated that human T1R3-NTD is functionally active and able to bind sucralose with a binding affinity in the millimolar range, which is in agreement with that measured with mouse T1R3-NTD [37, 41]. To investigate the structural basis of L-amino acid recognition by cat taste receptors, we used intrinsic tryptophan fluorescence signal to monitor ligand-binding to cT1R1-NTD. For this purpose, we expressed large quantities of recombinant cT1R1-NTD using the *E*. *coli* prokaryotic expression system as previously reported [35, 36]. SDS-PAGE analysis showed a band migrating at a molecular weight of approximately 55 kDa, in agreement with theoretical molecular mass values (55.4 kDa) calculated from the protein sequence. Soluble and insoluble proteins were separated by centrifugation and analysed using SDS-PAGE. As previously observed for human T1R2- and T1R3-NTDs, we observed that cT1R1-NTD was only detected in the insoluble fraction as inclusion bodies (S3 Fig). All attempts to produce soluble cT1R1-NTD by modifying expression conditions (i.e. IPTG concentration, induction time or temperature) were unsuccessful (S3 Fig). Electrophoresis analysis of purified cT1R1-NTD inclusion bodies showed the presence of a minor protein migrating at a molecular weight of approximately 31 kDa (Fig 2). Protein identification using peptide mass fingerprinting revealed that this protein corresponded to a N-terminal fragment of cT1R1-NTD. We found that induction of protein expression using 0.1 mM IPTG at 20°C for 3 hours reduced the proportion of this truncated form of cT1R1-NTD. cT1R1-NTD inclusion bodies were isolated and purified as previously described [35, 36]. From 1 litre of bacterial culture, we obtained approximately 150 mg of cT1R1-NTD. SDS-PAGE analysis of purified inclusion bodies revealed that cT1R1-NTD protein was more than 90% pure (S2B Fig). To increase the refolding yield of cT1R1-NTD, improvements were made to our previously reported protocol for hT1R3-NTD [35], which is described in S2A Fig. Briefly, cT1R1-NTD was refolded in the presence of a mixture of SDS and DDM, the SDS was removed using precipitation by KCl followed by dialysis. Isolation of the monomeric refolded protein was performed using preparative gel filtration chromatography. As shown in Fig 3, cT1R1-NTD eluted at approximately 13.75 mL with a purity, as indicated by SDS-PAGE analysis, of more than 95% (S2 Fig). The calibration of the gel filtration column with molecular mass markers confirmed the eluting position of the refolded cT1R1-NTD at a molecular weight of 57 kDa in agreement with a monomeric state of cT1R1-NTD. Western blot analysis using anti-his-tag antibodies confirmed that cT1R1-NTD was the main protein present in the sample (S2C Fig).

### Characterisation of cT1R1-NTD

**Fig 4:**
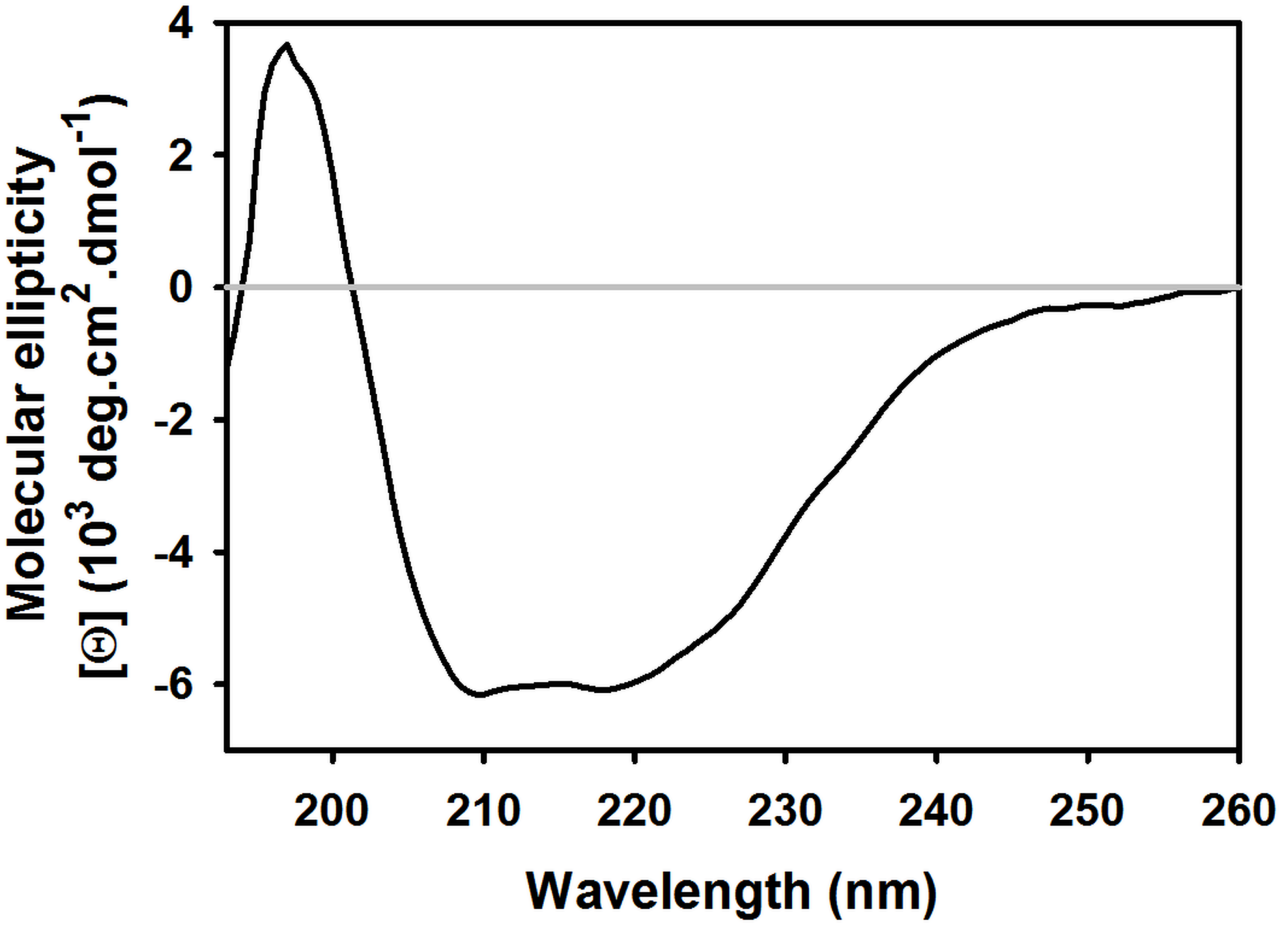
Characterization of cT1R1-NTD using far-UV circular dichroism spectroscopy. Protein concentration in 50 mM Tris-HCl pH 8.0, 150 mM NaCl, 1mM DTT and 0.1 mM DDM was approximately 0.2 mg/ml. Light path: 0.01 cm.

**Fig 5:**
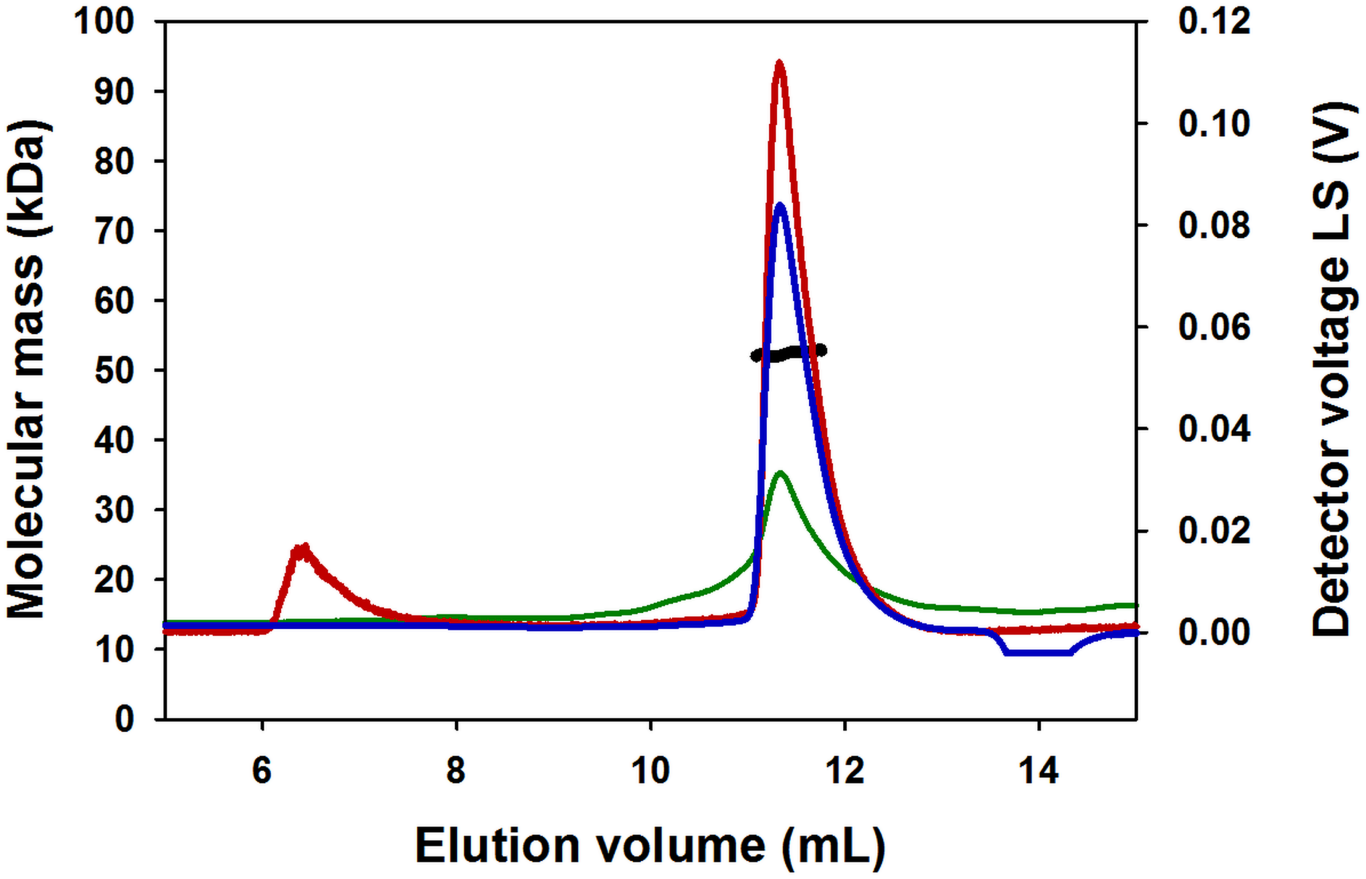
SEC-MALS analysis of cT1R1-NTD. The column was equilibrated and cT1R1-NTD eluted with 50 mM Tris-HCl pH 7.5, 150 mM NaCl, 0.1 mM DDM. The chromatograms show the readings of the light scattering (LS), the differential refractive index (dRI) and UV detectors in red, blue and green, respectively. The scale for the LS detector is shown in the right-hand axis. The thick black line indicates the calculated molecular mass of the eluting protein throughout the chromatogram (scale on the left-hand axis). cT1R1-NTD has a fitted molecular mass of 52.5 kDa; its theoretical monomer molecular mass value is 55.4 kDa.

To confirm the folding and structural integrity of refolded cT1R1-NTD, we used CD spectroscopy. The cT1R1-NTD far-UV spectrum displayed a positive peak centered at 193 nm and two negative peaks at 209 and 222 nm characteristic of a folded protein containing helical secondary structures (Fig 4). The deconvolution of the CD spectrum revealed that cT1R1–NTD was composed of approximately 27% α-helix and 20% β-sheet. This composition is consistent with that measured on mouse and human T1R2- and T1R3-NTDs [35, 37]. The fluorescence properties of the tryptophan residues in a protein could be used to probe the refolded state of proteins using denaturing chemicals. For this purpose, we recorded the fluorescence tryptophan spectra of refolded cT1R1-NTD in the absence and presence of 6 M guanidine hydrochloride (S4 Fig). A red-shift of 10 nm (340 to 350 nm) and a 50% increase in fluorescence intensity was observed revealing an increased exposure of tryptophan residues to the solvent. Both of these fluorescent variations confirmed that cT1R1-NTD was folded prior to denaturation. This observation demonstrates the overall structural homogeneity of the protein sample with respect to folding. To evaluate the oligomeric state of purified cT1R1-NTD, we used size-exclusion chromatography (SEC) in combination with on-line multiangle laser light scattering (MALS) and refractometry. SEC-MALS is valuable to determine the subunit stoichiometry or oligomeric state of proteins in their native form in solution. This technique measures the absolute molecular weight of a protein in solution independently from its shape, elution order or interaction with the column matrix. cT1R1-NTD has a theoretical mass of 55.4 kDa. As shown in Fig 5, SEC-MALS analysis showed that cT1R1-NTD mainly elutes as a monodisperse peak with a measured mass of 52.5 kDa, which is close to the monomer theoretical mass value, suggesting that the refolded protein behaves as a monomer in solution. SEC-MALS analysis revealed the presence of small amounts of aggregates in the sample, which were estimated to be less than 1.3% of the total refolded cT1R1-NTD.

### Ligand binding properties of cT1R1-NTD

**Fig 6:**
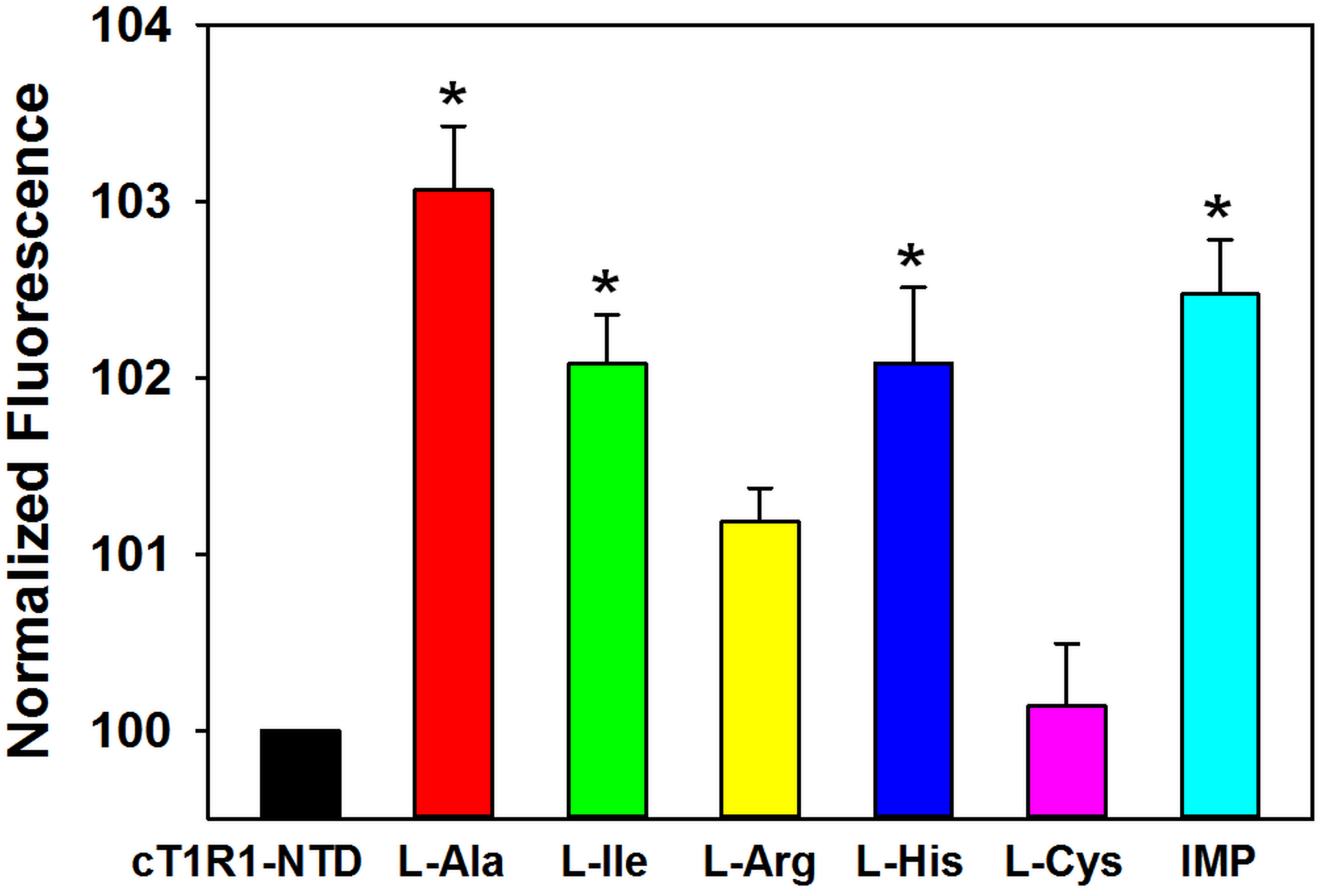
cT1R1-NTD binds L-amino acids and IMP. Normalized maximal fluorescence intensity of cT1R1-NTD before and after addition of ligands (100 μM final concentration). L-Cys does not affect cT1R1-NTD fluorescence. Fluorescence of cT1R1-NTD alone was defined as 100% in absence of ligand. Excitation and emission wavelength were 295 nm and 340 nm, respectively. cT1R1-NTD concentration was 0.5 μM. Data values are the mean ± SEMs of more than nine independent replicates of at least three independently refolded protein samples. *, Significantly different from cT1R1-NTD before addition of ligands (one-way ANOVA followed by Dunnett’s, p ≤ 0.05; for L-Arg p ≤ 0.08).

**Fig 7:**
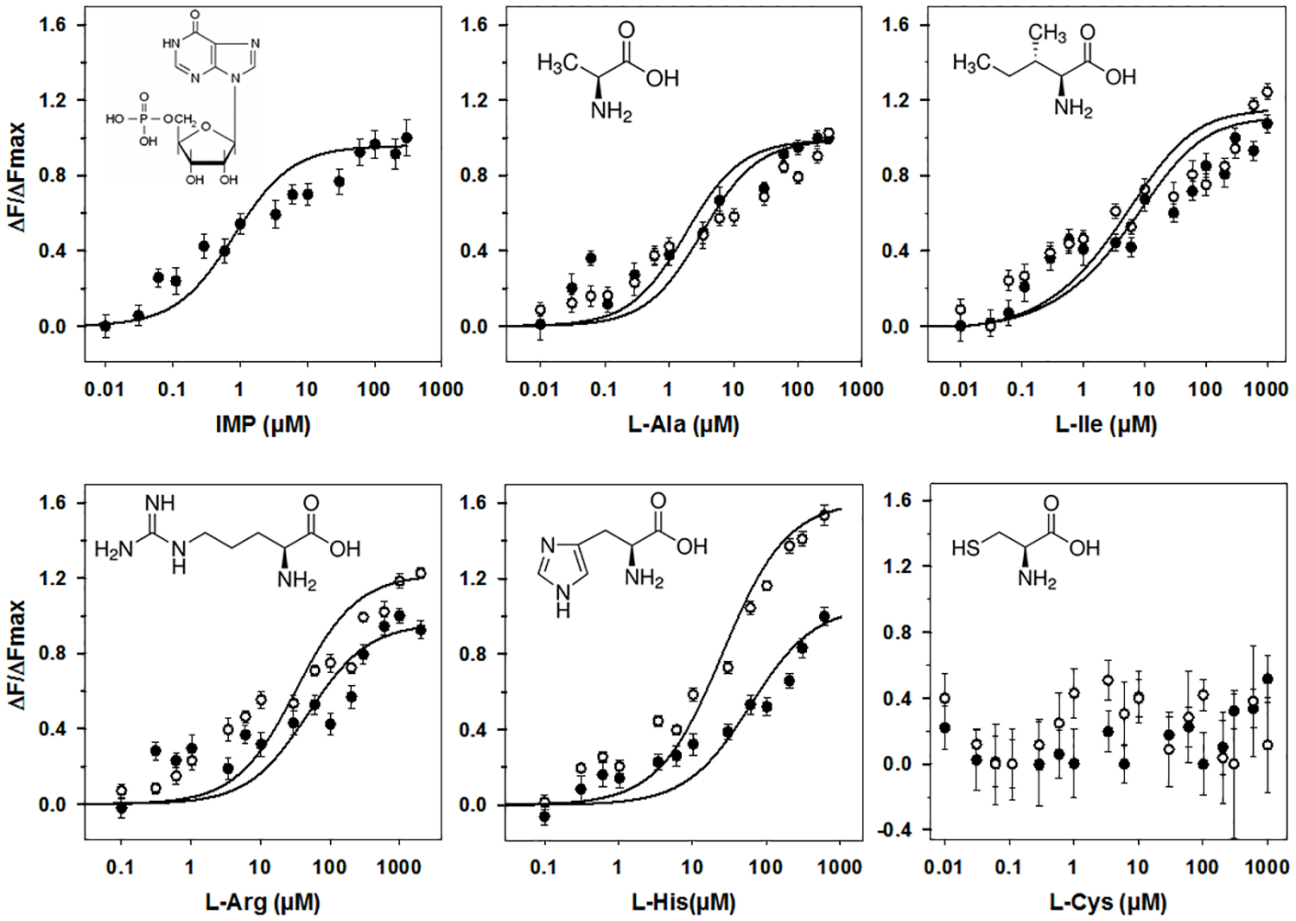
Binding properties of cT1R1-NTD assessed by intrinsic tryptophan fluorescence. Titration curves of cT1R1-NTD with IMP alone and L-amino acids in the presence (open circles) or absence of 0.3 μM IMP (black circles). Circles show experimental data, while the solid lines are the computed binding curves; excitation and emission wavelength were 295 nm and 340 nm, respectively; cT1R1-NTD concentration 0.5 μM. Fluorescence of cT1R1-NTD alone was defined as 100% in absence of ligand. Data points correspond to mean ± SEMs of more than nine independent replicates of at least three independently refolded protein samples. Data fitted with sigmoid dose-response curves using SigmaPlot software.

Cat behavioral experiments and molecular modeling studies have suggested that cat T1R1/T1R3 functions as a broadly tuned L-amino acid receptor [26]. Therefore, we measured the binding affinities of cT1R1-NTD for five L-amino acids belonging to different classes, including aliphatic (L-Ala, L-Ile,), polar (L-Cys), and basic (L-His, L-Arg) amino acids for which, cats have different taste preferences [26]. For this purpose, we determined the concentration-response relationships for the intrinsic tryptophan fluorescence of cT1R1-NTD upon the addition of these representative ligands. Because the fluorescence emitted by tryptophan residues is highly sensitive to local environment changes, this technique allows the measurement of protein-ligand interaction in the absence of protein or ligand labeling. This label-free technique has been used to measure protein-ligand binding interaction with class C GPCR NTDs [36, 37, 41–43]. As shown in Fig 6, we found that the L-amino acids, L-Ala, L-Ile, L-Arg and L-His, induced conformational changes in cT1R1-NTD leading to a saturable intrinsic fluorescent enhancement. Titration curves revealed that L-Ala and L-Ile were the ligands with highest affinity, exhibiting *K*_d_ values of 3.2 ± 0.3 and 7.3 ± 0.7 μM respectively (Fig 7 and Table 1). In the case of L-Ile, it should be pointed out that at the high concentrations, the binding curve does not clearly form a plateau phase. This lack of plateau could be attributed to the poor aqueous solubility of L-Ile at the highest concentrations. We observed that L-Arg and L-His bound cT1R1-NTD with a lower affinity with *K*_d_ values >50 μM. This interaction is specific since the sulfur-containing amino acid (L-Cys) has no effect on the intrinsic fluorescence of cT1R1-NTD (Figs 6 and 7).

**Table 1:** Dissociation constants (*K*_d_) values of ligands for cT1R1-NTD.

Ligand	*K*_d_ [μM] No IMP	*K*_d_ [μM] 0.3 μM IMP
IMP	0.83 ± 0.06	-
L-Ala	3.2 ± 0.3	1.8 ± 0.1
L-Ile	7.3 ± 0.7	4.9 ± 0.6
L-Arg	48.1 ± 1.7	32.9 ± 4.1
L-His	83.4 ± 10.7	20.9 ± 1.4

The *K*_d_ values are reported as the mean ± SEM.

Robust synergism between L-Glu and the 5’-monophosphate ribonucleotides IMP and GMP is a hallmark of umami taste in humans and in many animal species [9, 44–46]. Since cats are responsive to IMP and GMP alone [31], we tested these compounds for their ability to affect cT1R1-NTD intrinsic fluorescence. Interestingly, the addition of IMP in the absence of amino acids resulted in an increase of cT1R1-NTD intrinsic fluorescence (Fig 6) leading to a *K*_d_ value of 0.83 ± 0.06 μM (Fig 7 and Table 1). Upon the addition of GMP alone at low concentrations (below 1 μM) we also measured a fluorescence enhancement, further demonstrating an interaction of this 5’-ribonucleotide with the cT1R1-NTD. Unfortunately, at higher GMP concentrations, we observed a strong decrease of fluorescence due to absorbance of the UV light by this 5’-monophosphate ribonucleotide impairing affinity determination. We then investigated the synergy between L-amino acids and IMP. As expected, low doses of IMP (0.3 μM IMP, final concentration) shifted the binding curves to the left for all tested amino acids (Fig 7). However, we found this synergetic effect was more pronounced for L-His and relatively modest for L-Ala, L-Ile, and L-Arg (Table 1). Interestingly, we noted that the presence of 0.3 μM IMP increased the maximal amplitude of cT1R1-NTD fluorescence induced by L-Arg and L-His, compared to the other tested L-amino acids (Fig 7). The enhancement effect of IMP was specific since the fluorescence enhancement of cT1R1-NTD generated by 0.3 μM IMP in the presence of L-Cys was not significantly different from that observed in the absence of L-Cys (Fig 7 and S5 Fig). Taken together, these results clearly indicate that cT1R1-NTD isolated from inclusion bodies is functional after re-folding and suggest that cat T1R1/T1R3 receptor is broadly activated by many L-amino acids. Finally, our data confirm that there is a synergy between amino acids and IMP involved the N-terminal Venus flytrap domain of T1R1.

## Discussion

The umami cat receptor subunits (T1R1 and T1R3) belong to class C of GPCRs and play an important role in the detection of L-amino acids, which is especially important for cat food choice as they are obligate carnivores. By combining *in silico* homology models of T1R1 and behavioural experiments, it was proposed that cat T1R1/T1R3 is a broadly-tuned amino acid receptor able to recognize eleven naturally-occurring amino acids in synergy with the ribonucleotides IMP and GMP [26].

Here we show that cT1R1-NTD can be produced as a functionally-active protein in milligram quantities. CD demonstrated that cT1R1-NTD was properly folded with the developed procedure and contained secondary structures that matched with those measured for refolded hT1R3-NTD [35] or those of mT1R2- and mT1R3-NTD produced in bacteria as soluble proteins [37, 41]. In contrast with hT1R3-NTD, which was observed to be a dimer by gel filtration [35], SEC-MALS analysis revealed that cT1R1-NTD exists as a monomer indicating that the protein domain is stable in the absence of its T1R3 binding partner. The oligomeric state of cT1R1-NTD is similar to that observed for the NTDs of GABA_B_ receptor heterologously expressed by insect cells. GABA_B_ is formed by the heterodimeric assembly of GBR1 and GBR2 subunits. GBR2-NTD was observed as a monomer, whereas GBR1-NTD was found to occur as a monomer-dimer equilibrium [47]. Recently, Neumket et al [14] solved the crystal structure of the medaka fish T1R2/T1R3 heterodimer. The structure revealed that Cys132 located in loop 2 of T1R3 and Cys344 located in loop 3 of T1R2 forms an inter-subunit disulphide bridge, which is a key for the heterodimer stabilization. In the case of homodimeric calcium-sensing receptor (CaSR) and mGluR1, cysteine residues located in loop 2 form intermolecular bridge(s) between subunits [13, 48]. Since cat T1R1 has no cysteine residues in loop 2, but does have a cysteine residue present in loop 3 (S1 Fig), no disulphide bridges are expected between cT1R1 monomers, in agreement with the measured monomeric state of cT1R1-NTD.

Our intrinsic fluorescence data demonstrated that recombinant cT1R1-NTD was able to bind various L-amino acids suggesting that cat T1R1/T1R3 is a broadly tuned amino acid receptor as previously reported for the mouse T1R1/T1R3 [8, 15]. We measured affinities in the micromolar range with dissociation constant values lower than predicted values deduced from behavioural experiments conducted on cats [26] or half maximal effective concentration (EC_50_) values from mouse or human T1R1/T1R3 receptor measured using a calcium-based cellular assay [8, 15, 49, 50]. Interestingly, Nie et al (2005) have already reported slightly lower *K*_d_ values for mouse T1R2-NTD compared to predicted values deduced from rodent behavioural studies or receptor functional assays [37]. Possible explanations could be a low coupling efficiency of this receptor to the artificially created signalling cascade in the cellular assay or the impact of receptor heterodimerization on receptor sensitivity. Thus, it has been observed that the interaction of GBR1-NTD with GBR2-NTD affects the affinity of GBR1-NTD for agonist [47]. Our study showed that L-Ala and L-His bind to cT1R1-NTD in agreement with the clear preference of cats for water supplemented with these amino acids [26]. We observed that cT1R1-NTD also bound the negatively charged amino acid, L-Arg although cats do not show a preference for this compound in solution [26]. The cat aversion for L-Arg can be explained by the high pH value of the non-buffered amino acid solutions known to be aversive for cats [24]. This hypothesis could also explain the discrepancy of cat preference observed for instance for L-Lys solution [26, 27]. In addition, the possibility of a negative off-taste as bitter, generated by L-Arg could not be excluded [24].

Conversely, we observed that the sulphur-containing L-Cys, a palatable amino acid for cats, does not interact with cT1R1-NTD [26] whereas this amino acid was reported to be a strong agonist of mouse T1R1/T1R3 [51]. The absence of L-Cys binding onto cT1R1-NTD could be explained by species-dependent differences or more probably the presence of additional cat umami receptors as proposed in rodents [16–21]. However, as pointed out by Toda et al. [15], we cannot exclude the problem of L-Cys chemical instability in our assay buffer. Although the Venus flytrap domain of T1R1 has been demonstrated to be responsible for amino acid and IMP recognition [11, 12, 15], we cannot fully exclude the possibility that T1R3-NTD participates in the binding of some amino acids or modifies the binding specificity of T1R1-NTD. Thus, mouse and human T1R3-NTD have been shown to bind sweet molecules such as natural sugars and sucralose [35, 37]. The amino acid binding properties of cat T1R3-NTD should be clarified in future studies.

Importantly, our data revealed that the L-amino acid binding onto cT1R1-NTD is enhanced by low doses of IMP (0.3 μM) further demonstrating that cT1R1-NTD is properly refolded and functional. This observation is in agreement with the molecular mechanism of synergy demonstrated with human T1R1/T1R3 umami receptor in which, IMP binds to close to the hinge region of T1R1-NTD, and IMP bind to an adjacent site stabilizing the NTD of T1R1 in its closed conformation [12]. Our study revealed that the synergistic effect of IMP depends on the nature of the L-amino acids, in good agreement with functional studies on mouse T1R1/T1R3 [8, 15]. We also observed that the presence of IMP enhanced the maximal fluorescence amplitude of cT1R1-NTD for some L-amino acids such as L-His and L-Arg. We can speculate that the synergistic effect of IMP and these amino acids leads to a different conformation change of cT1R1-NTD, which could affect the efficacy of these ligands to activate the cat T1R1/T1R3 receptor. This hypothesis could be validated by combining biophysical approaches, site-directed mutagenesis and functional expression studies conducted on the full-length receptor. Additionally, our data provide evidence through direct binding interaction that IMP binds cT1R1-NTD in the absence of L-amino acids. In humans, it is still controversial if 5’-ribonucleotides, IMP and GMP generate an umami taste by themselves or synergise with the low concentration of free L-Glu naturally present in human saliva [9, 52]. Our results strongly suggest that cat T1R1/T1R3 receptor is activated by IMP in the absence of amino acids encouraging us to speculate that a similar phenomenon may occur in the human umami receptor.

In summary, our study demonstrated that cT1R1-NTD is able to bind L-amino acids with *K*_d_ values in the micromolar range and compatible with values predicted from cat behavioural experiments. In addition, we demonstrated a positive allosteric effect of the 5’-monophosphate ribonucleotide, IMP on L-amino acid binding, which is the hallmark of umami taste. Our approach will be valuable to produce a large amount of purified proteins required for structural studies including NMR spectroscopic analyses or crystallization trials. Finally, it offers an interesting new experimental strategy to screen new umami tastants or umami enhancers.

## Supporting information

S1 Fig Amino acid sequence of cT1R1-NTD. cT1R1-NTD sequence (Leu21-Ser495) is in green. Numbers refer to amino acid residues of cT1R1 (Signal peptide: 1–20). His-Tags and thrombin cleavage site are shown in yellow and pink, respectively. The cysteine residue located in loop 3 expected to form a disulphide bridge between cT1R1 and cT1R3 subunits is shown in grey.
(TIF)

S2 Fig Production of pure refolded cT1R1-NTD. (A) Schematic summary of key steps in the production of pure refolded cT1R1-NTD. Blue numbers on the left indicate samples analyzed by SDS-PAGE (B) and western-blot analysis using anti-his-tag antibodies (C). The proteins were separated by 4–15% SDS-PAGE and stained with Coomassie blue. The molecular mass markers are in lane M. Presence of cT1R1-NTD was probed using Western-blot analysis. Proteins were separated by 4–15% SDS-PAGE followed by electroblotting to a PVDF membrane using the mouse anti-his monoclonal antibody (1:2,000) and HRP-conjugated goat antimouse IgG (1:50,000) as a secondary antibody.
(TIF)

S3 Fig SDS-PAGE analysis of cT1R1-NTD expressed in bacteria. *E*. *coli* BL21 (DE3) cells were transformed with pET28-cT1R1-NTD before and after IPTG induction for 1, 2 and 3 hours. Soluble and insoluble protein fractions (inclusion bodies) were separated and loaded onto 12% SDS-polyacrylamide gel with size control of molecular mass markers (lane M). Proteins were stained with Coomassie blue. Position of cT1R1-NTD is indicated by an arrow.
(TIF)

S4 Fig Characterization of cT1R1-NTD folding. cT1R1-NTD intrinsic tryptophan fluorescence in the absence (solid line) and presence of 6M guanidine hydrochloride (GuCl) (dashed line). Excitation wavelength 295 nm and temperature of the cuvette was maintained at 20°C.
(TIF)

S5 Fig IMP did not enhance binding of L-Cys onto cT1R1-NTD. Normalized maximal fluorescence intensity of cT1R1-NTD in the presence and absence of L-amino acids (100 μM final concentration). L-Cys does not affect cT1R1-NTD fluorescence. Fluorescence of cT1R1-NTD alone was defined as 100% in absence of ligand. Excitation and emission wavelength were 295 nm and 340 nm, respectively. cT1R1-NTD concentration was 0.5 μM. Data values are the means ± SEMs of more than nine independent replicates of at least three independently refolded protein samples. *, Significantly different from tastant L-Cys (one-way ANOVA followed by Dunnett’s, p ≤ 0.05).
(TIF)
