# Supplementary figures and images for: Biophysical and functional characterization of the N-terminal domain of the cat T1R1 umami taste receptor expressed in Escherichia coli
Source: PLoS One. 2017 Oct 30;12(10):e0187051. doi: 10.1371/journal.pone.0187051 (PMC5662223; doi:10.1371/journal.pone.0187051)

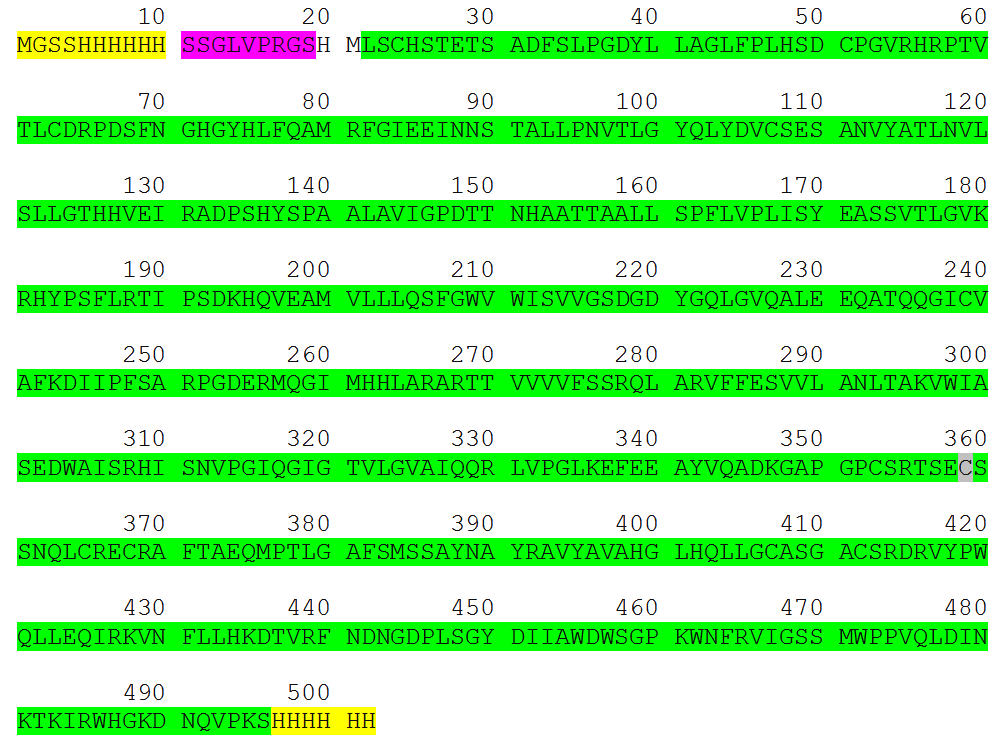

Supplement: S1 Fig — cT1R1-NTD sequence (Leu21-Ser495) is in green. Numbers refer to amino acid residues of cT1R1 (Signal peptide: 1–20). His-Tags and thrombin cleavage site are shown in yellow and pink, respectively. The cysteine residue located in loop 3 expected to form a disulphide bridge between cT1R1 and cT1R3 subunits is shown in grey. (TIF) [file pone.0187051.s001.tif]

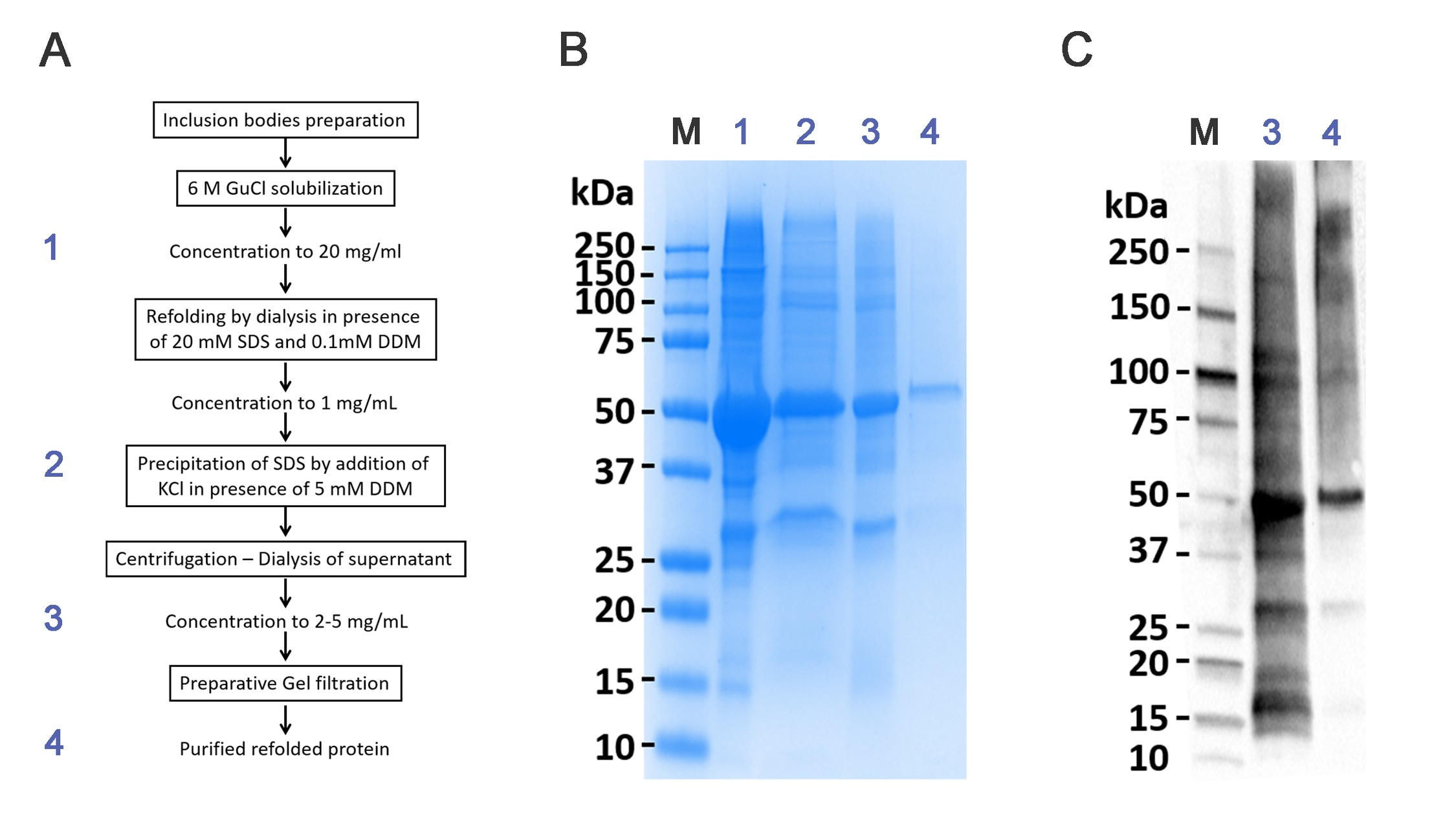

Supplement: S2 Fig — (A) Schematic summary of key steps in the production of pure refolded cT1R1-NTD. Blue numbers on the left indicate samples analyzed by SDS-PAGE (B) and western-blot analysis using anti-his-tag antibodies (C). The proteins were separated by 4–15% SDS-PAGE and stained with Coomassie blue. The molecular mass markers are in lane M. Presence of cT1R1-NTD was probed using Western-blot analysis. Proteins were separated by 4–15% SDS-PAGE followed by electroblotting to a PVDF membrane using the mouse anti-his monoclonal antibody (1:2,000) and HRP-conjugated goat antimouse IgG (1:50,000) as a secondary antibody. (TIF) [file pone.0187051.s002.tif]

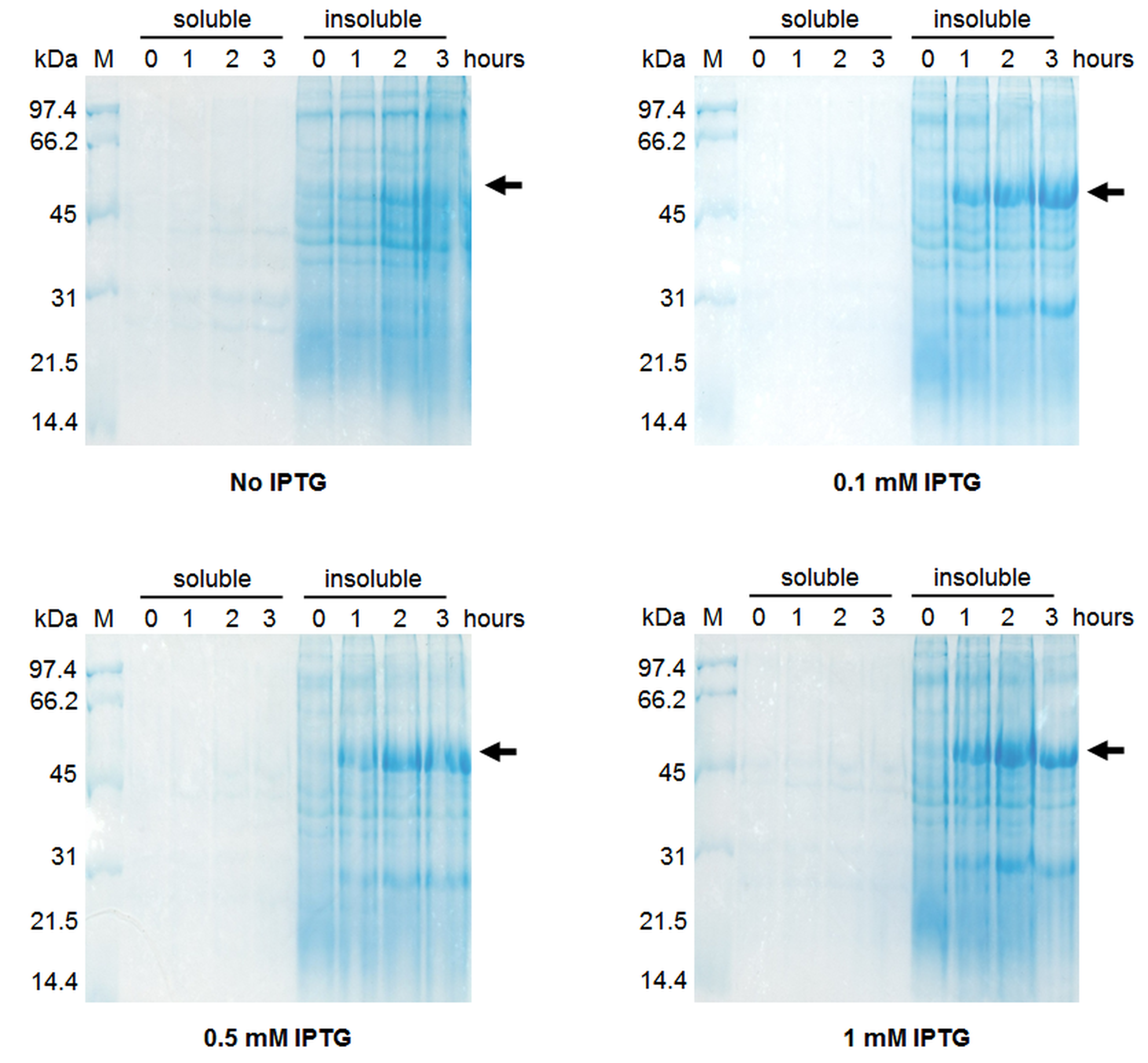

Supplement: S3 Fig — E. coli BL21 (DE3) cells were transformed with pET28-cT1R1-NTD before and after IPTG induction for 1, 2 and 3 hours. Soluble and insoluble protein fractions (inclusion bodies) were separated and loaded onto 12% SDS-polyacrylamide gel with size control of molecular mass markers (lane M). Proteins were stained with Coomassie blue. Position of cT1R1-NTD is indicated by an arrow. (TIF) [file pone.0187051.s003.tif]

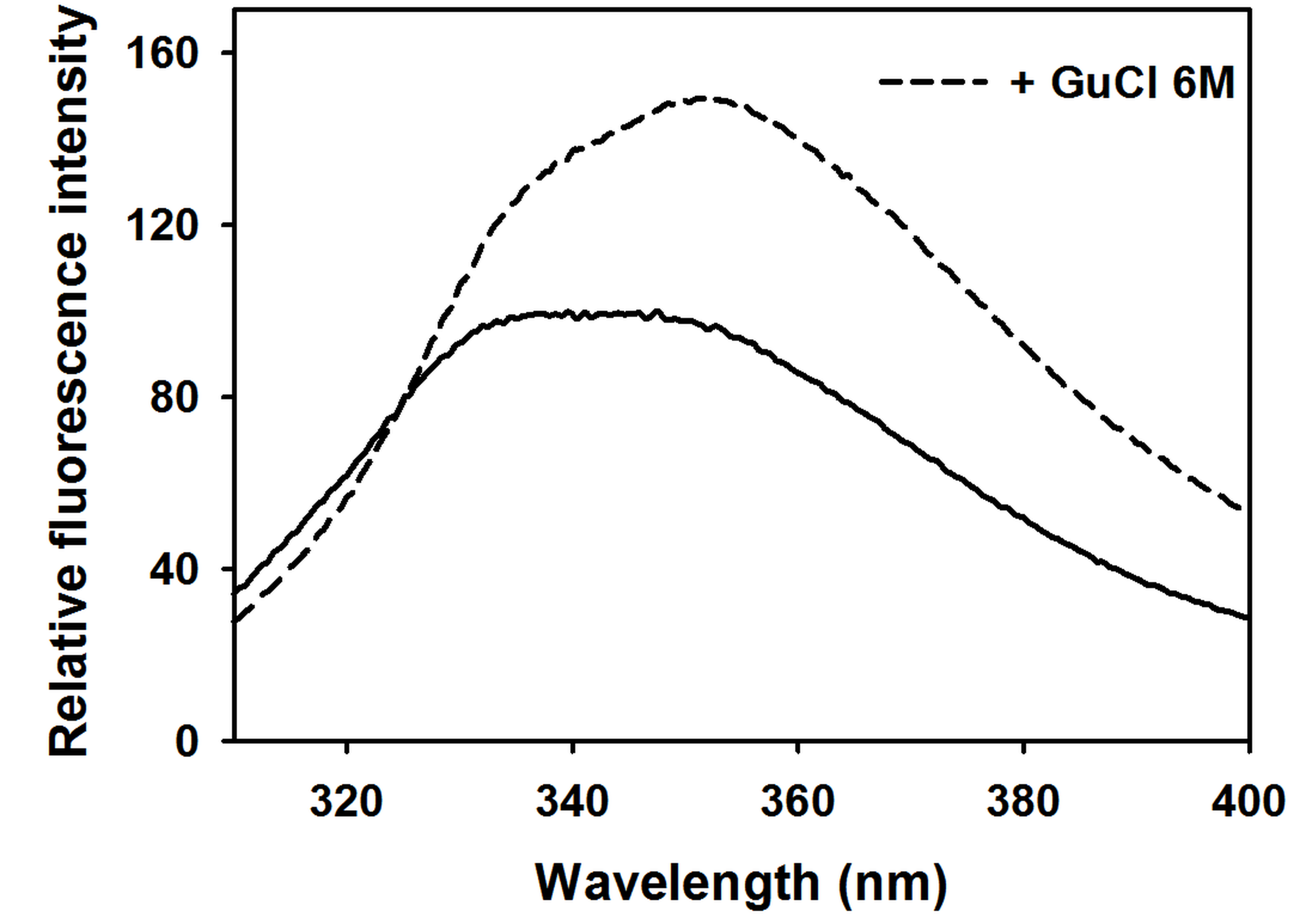

Supplement: S4 Fig — cT1R1-NTD intrinsic tryptophan fluorescence in the absence (solid line) and presence of 6M guanidine hydrochloride (GuCl) (dashed line). Excitation wavelength 295 nm and temperature of the cuvette was maintained at 20°C. (TIF) [file pone.0187051.s004.tif]

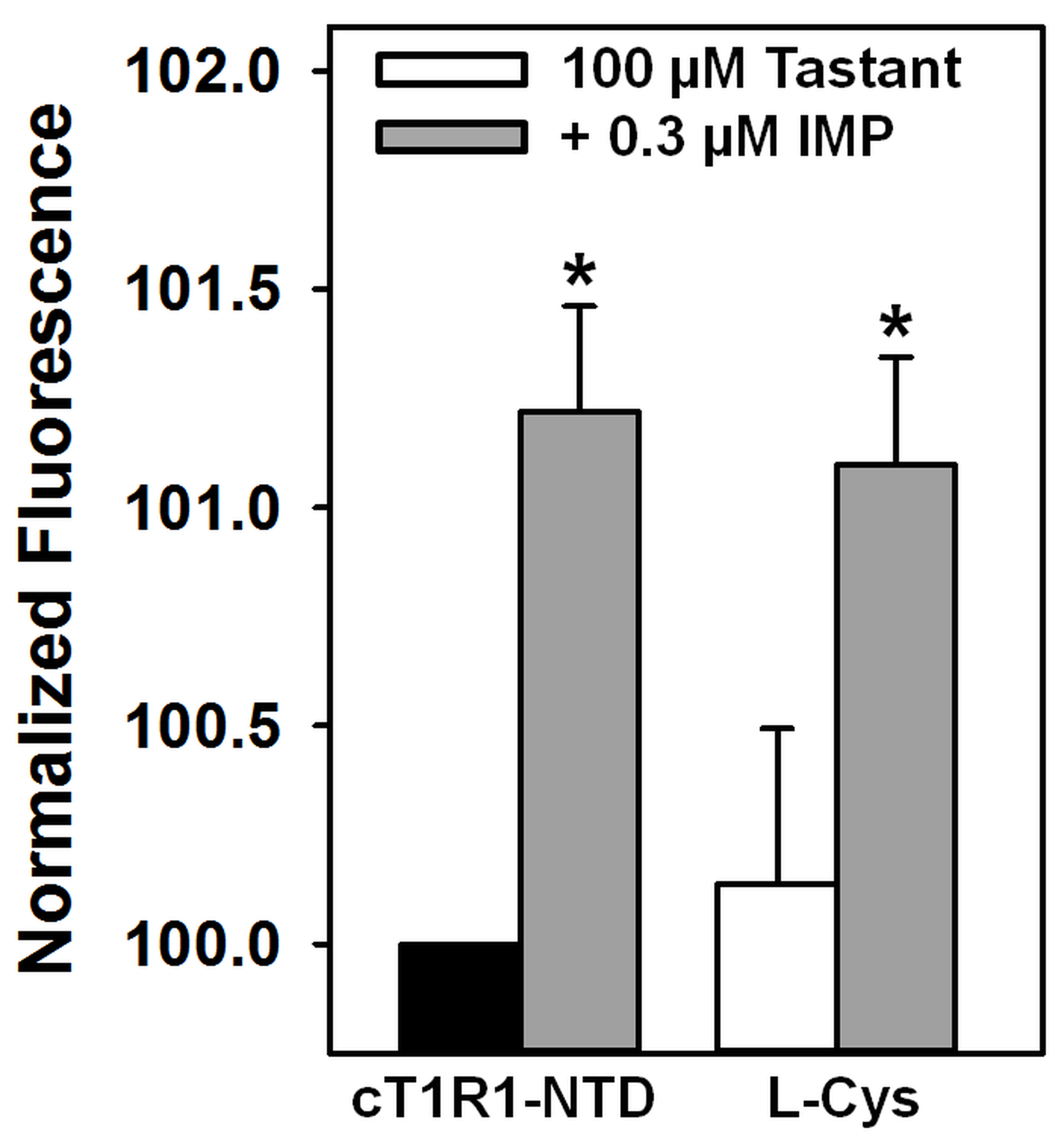

Supplement: S5 Fig — Normalized maximal fluorescence intensity of cT1R1-NTD in the presence and absence of L-amino acids (100 μM final concentration). L-Cys does not affect cT1R1-NTD fluorescence. Fluorescence of cT1R1-NTD alone was defined as 100% in absence of ligand. Excitation and emission wavelength were 295 nm and 340 nm, respectively. cT1R1-NTD concentration was 0.5 μM. Data values are the means ± SEMs of more than nine independent replicates of at least three independently refolded protein samples. *, Significantly different from tastant L-Cys (one-way ANOVA followed by Dunnett’s, p ≤ 0.05). (TIF) [file pone.0187051.s005.tif]
